# 

**Published:** 1908-09-12

**Authors:** 


					0/
f >1
1 A aJ
Talagr&phlo Address i
Ospedale, London.
THE MODERNf ind< 'x^^ph^ne N?"?ben
MEDICAL JOURNAL. s ?" " "" Gm"d'
*EW SERIES. No. 79, Vol. III. Qatttrhav
No. 1151. Vol. XLIV. SATURDAY,
Sept. 12th, 1908.
PRICE THREEPENCE

				

